# Effects of Multimodal Analgesic Protocol, with Buprenorphine and Meloxicam, on Mice Well-Being: A Dose Finding Study

**DOI:** 10.3390/ani11123420

**Published:** 2021-11-30

**Authors:** Kayo Furumoto, Kumi Ogita, Tomomi Kamisaka, Asami Kawasumi, Koushi Takata, Noritaka Maeta, Takamasa Itoi, Masakatsu Nohara, Kaori Saeki, Teppei Kanda

**Affiliations:** 1 College of Life Science, Kurashiki University of Science and the Arts, Tsurajima-cho Nishinoura 2640, Kurasiki 7128555, Okayama, Japan; k-furumoto@vet.ous.ac.jp (K.F.); kurosun1020@gmail.com (K.O.); love.koglo@gmail.com (T.K.); pwj655@yahoo.co.jp (A.K.); takatakoshi0001@gmail.com (K.T.); n-maeta@vet.ous.ac.jp (N.M.); t-itoi@vet.ous.ac.jp (T.I.); 2 Faculty of Veterinary Medicine, Okayama University of Science, Ikoino-oka 1-3, Imabari 7948555, Ehime, Japan; m-nohara@vet.ous.ac.jp (M.N.); k-saeki@vet.ous.ac.jp (K.S.)

**Keywords:** pain assessment, multimodal analgesics, distress, well-being, buprenorphine, meloxicam, mouse

## Abstract

**Simple Summary:**

Inadequate pain management affects animal welfare and scientific data validity. Multimodal analgesia is effective in reducing postoperative pain. However, surgery-related pain is not the only distress animals suffer during the perioperative period. The anesthetic or analgesic agent of choice, route and frequency of anesthetic or analgesic administration, and stressors such as anxiety and fear also induce distress. We hypothesized that a multimodal analgesic protocol using buprenorphine and meloxicam would have analgesic effects, and evaluated the effects of methods and drugs used for anesthesia and analgesia on the well-being of mice assigned to different groups. Even in the absence of surgical pain, the anesthesia + analgesia group presented the same negative effects as the surgery + anesthesia + analgesia group. The multimodal analgesic protocol, using buprenorphine and meloxicam, for mice is expected to have an analgesic effect on pain associated with laparotomy but was not sufficient in preventing food intake and weight decrease. This does not negate the need to administer analgesics, but suggests the need to focus on and care not only about the approach to relieve pain associated with surgery, but also other types of distresses in order to minimize negative side effects that may interfere with postoperative recovery in mice.

**Abstract:**

The anesthetic or analgesic agent of choice, route and frequency of anesthetic or analgesic administration, and stressors induce distress during the perioperative period. We evaluated a multimodal analgesic protocol using buprenorphine and meloxicam on the well-being of mice. Twenty-four Slc:ICR male mice were divided into control, anesthesia + analgesia, and surgery + anesthesia + analgesia groups. Tap water (orally: PO) and water for injection (subcutaneous: SC) were administered to the control group. Buprenorphine was administered twice (SC, 0.1 mg/kg/8 h) and meloxicam was administered thrice (PO, 5 mg/kg/24 h) to the anesthesia + analgesia and surgery + anesthesia + analgesia groups. The mice were subjected to laparotomy and assessed for several parameters. Even in absence of surgical pain, the anesthesia + analgesia group presented the same negative effects as the surgery + anesthesia + analgesia group. This multimodal analgesic protocol for mice was expected to have an analgesic effect on pain associated with laparotomy but was not sufficient to prevent food intake and weight decrease. This does not negate the need to administer analgesics, but suggests the need to focus on and care not only about the approach to relieve pain associated with surgery, but also other types of distresses to minimize negative side effects that may interfere with postoperative recovery in mice.

## 1. Introduction

Even if an animal feels pain, it does not always express it. The Institute for Laboratory Animal Research Guide for the Care and Use of Laboratory Animals states “it should be assumed that procedures that cause pain in humans also cause pain in animals” [1]. Pain induces distress and has negative physiological consequences in animals, affecting not only animal welfare but also scientific data validity [2]. Appropriate perioperative pain management enhances the safety of anesthesia and the effect of postoperative pain management. “Preemptive analgesia”, in which analgesics are administered before applying pain stimuli, and “multimodal analgesics”, wherein multiple analgesics with different mechanisms of action are used, minimize postoperative stress in animals and optimize the well-being of animals [3]. The pain associated with surgery is not the only distress animals suffer during the perioperative period. The anesthetic or analgesic agent of choice, route and frequency of administration of the anesthetic or analgesic agent also induce pain, and stressors such as anxiety and fear affect the pain perception. Repeated injections of drugs and forced oral administration involve restraining the animals, which may cause additional stress and increase existing pain [2]. The choice of a drug, route of administration, and frequency of administration should be considered when applying multiple drugs with different durations of action and pharmacokinetics for appropriate pain management.

Although a single analgesic is commonly used for postoperative pain management in mice, it does not exert sufficient analgesic effect [4,5,6]. The multimodal approach is not commonly used in postoperative pain management in rodents. While there are reports of positive effects for postoperative pain management [5,7], there are also reports of single analgesics being more effective [8], or having negative effects [9], and reports on their efficacy, adverse effects, and effects on animal well-being are not enough [2].

Buprenorphine and meloxicam are commonly used analgesics. Buprenorphine is a long-acting opioid used in postoperative pain management in rodents [8,10]. Meloxicam is a long-acting NSAID that requires once-daily administration [10]. These two agents were selected in our study since reducing the frequency of analgesic administration also contributes to reducing distress and pain in animals [2].

Here, we hypothesized that a multimodal analgesic protocol using buprenorphine and meloxicam would have analgesic effects. The purpose of our study was to evaluate the effects of anesthetics and analgesics used and the methods employed for their administration (administration techniques) on the well-being of mice. Body weight, food intake, body temperature, and animal behavior were evaluated. Burrowing and nest building behaviors, which are innate behaviors, have been used to evaluate the well-being of mice as previously reported [11,12,13]. Furthermore, these behavioral evaluations have been considered effective for evaluating pain in postoperative mice in cages [2,5,14,15,16,17,18].

## 2. Materials and Methods

### 2.1. Animals and Experimental Setup

**Figure 1:**
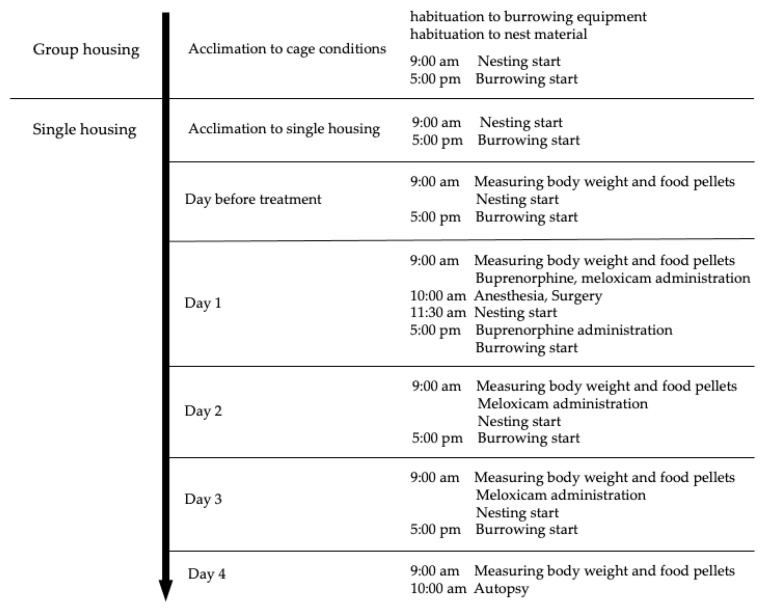
Outline of experimental schedule.

Twenty-four male mice (Slc:ICR: 5–7 weeks) were obtained from SLC (Hamamatsu, Japan) and used in this study. The animal room was maintained at a temperature of 24–26 °C and relative humidity of 40–60%, under a 12 h light (8:00–20:00 h) and 12 h dark (20:00–8:00 h) cycle with an artificial light. Mice were housed in individually ventilated cages (NIKI SHOUJI Co., Tokyo, Japan) and fed a pelleted diet (CE-2, CLEA Japan, Inc., Tokyo, Japan) and water *ad libitum*. A clear plastic cage with a floor area of 259 mm × 476 mm was used, and 60 g of wood shavings (CL-4161, CLEA Japan, Inc.) was used as bedding material. The food pellets were placed on the floor in a small pottery plate. The water bottle was set on the wire mesh. A water bottle (CL-2707, Japan Claire Co., Ltd., Tokyo, Japan) containing burrowing substrate (140 ± 2 g) and 5 g of nesting material (Enviro-dri; Shepherd Specialty Papers) was set on the floor of the cage. Flat glass pellets (diameter 20 mm) were used as the burrowing substrate. Three mice were housed in the same cage for the first seven days of acclimatization, and each mouse was housed in individual cages for the other seven days. Figure 1 shows the outline of the experimental schedule. The Animal Care and Use Committee of Kurashiki University of Science and the Arts approved this study (approval number, 2018-06).

### 2.2. Surgery and Perioperative Care

One hour before the start of anesthesia, the mice were subcutaneous injected 0.1 mg/kg buprenorphine (Nissin Pharmaceutical Co., Ltd., Yamagata, Japan) subcutaneously and administered 5 mg/kg meloxicam (Boehringer Ingelheim Vetmedica Japan Co., Ltd., Tokyo, Japan) orally. Later, 0.2 mg/kg medetomidine (Kyoritsu Seiyaku Co., Tokyo, Japan) was intraperitoneally injected 20 min before the induction of anesthesia. A 27 gauge needle (NIPRO Corporation, Osaka, Japan) was used for subcutaneous injection, a 26 gauge needle (NIPRO Corporation, Osaka, Japan) was used for intraperitoneal injection, and a 20 gauge oral gavage needle (Natsune Seisakusho Co., Ltd., Tokyo, Japan) was used for oral administration. After checking sedation caused by medetomidine, the vaporizer was set to 2.5% (100% O_2_; flow rate 1000 mL/min), and the mice were anesthetized with isoflurane (DS Pharma Animal Health Co., Ltd., Osaka, Japan) in an induction chamber (RC2 Rodent Circuit controller Anesthesia System; VetEquip Inc., Livermore, CA, USA) until the righting reflex was lost. The vaporizer was then set to 1.5% to 2.0% (100% O_2_; flow rate 500 mL/min), and general anesthesia was maintained via nose cone until the end of surgery. The hair at the surgical site in the lower abdomen was shaved, the skin in the surgical field was disinfected, and the eyes were covered with artificial tears (Senju Pharmaceutical, Osaka, Japan). After confirming the loss of pedal withdrawal, the mice were subjected to laparotomy. The midline skin and muscle (rectus abdominis), approximately 1 cm head-side from the penis, were subjected to a 1.5 cm incision. The testis, epididymis, and deferens duct were exposed outside the body to mimic vas deferens ligation surgery and then returned to the abdominal cavity after 3 min. The procedure was performed bilaterally. Thereafter, a small temperature-measuring device (nano tag; KISSEI COMTEC Co., Ltd., Nagano, Japan) was implanted into the body, and the muscle and skin were closed. The muscle was closed with a suture and the skin was closed using skin staples. Before closing the skin, a few drops of 0.5% bupivacaine (AstraZeneca K.K., Osaka, Japan) were dropped on the sutured rectus abdominis area. After completing the surgery, the mice were intraperitoneally injected 1 mg/kg atipamezole (Kyoritsuseiyaku Co., Tokyo, Japan). All surgeries were performed by the same experimenter. After atipamezole administration, the righting reflex of the mice was confirmed, and they were placed on a warming plate for 30 min, and then returned to the cages in the animal room. The mice were kept warm on the warming plate from the start of maintenance anesthesia until they were returned to the cages in the animal room after surgery. The mice were subcutaneous injected 0.1 mg/kg buprenorphine, 8 h after the first administration. In addition, they were administered 5 mg/kg meloxicam orally at 9:00 a.m. on days 2 and 3.

### 2.3. Treatment Groups

The mice were randomized into three groups with eight mice per group: (1) the “control” group, (2) the “anesthesia + analgesia” group, and (3) the “surgery + anesthesia + analgesia” group. In the control group, without anesthesia and surgery, the mice were administered water for injection instead of buprenorphine, medetomidine, and atipamezole and water instead of meloxicam. In the anesthesia + analgesia group, without surgery, the mice were anesthetized and treated with analgesics (no treatment for bupivacaine). In the surgery + anesthesia + analgesia group, the mice were anesthetized, treated with analgesics, and operated upon (Table 1).

### 2.4. Data Collection

#### 2.4.1. Food Intake

Three diets were placed on the floor in a small pottery plate and changed daily at 9:00 a.m. Food intake was recorded at 24 h intervals from the day before treatment to day 4. Food intake was determined as the difference in the weight of food 24 h after and before placing the food in the cage. Food intake 24 h before treatment was used as the baseline value (the value at 9:00 a.m. on day 1 minus the value at 9:00 a.m. on the day before treatment).

#### 2.4.2. Body Weight

The body weight of mice was recorded daily at 9:00 a.m. From the day one to day four, body weight was recorded at 24 h intervals. The body weight just before the treatment was recorded as the baseline value (the value at 9:00 a.m. on day 1).

#### 2.4.3. Nest Consolidating Behavior

**Figure 2:**
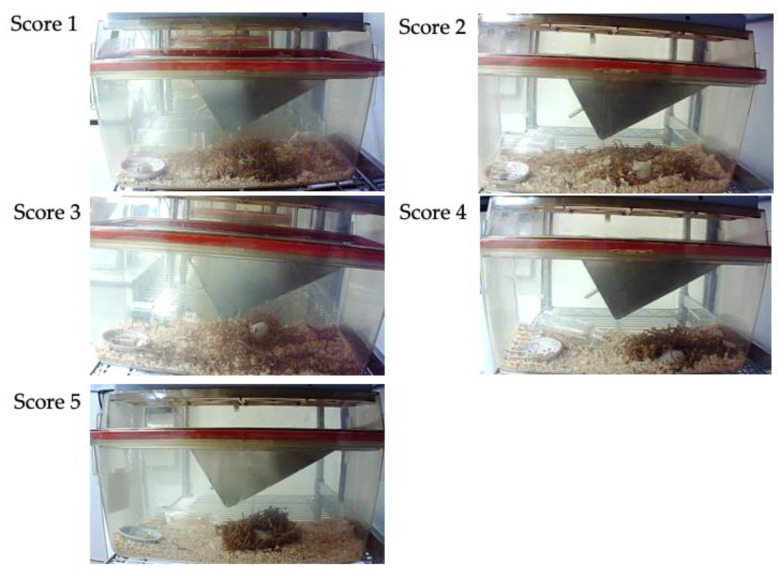
Nest consolidation scores.

Mice nest consolidating behaviors were recorded using a network camera (Qwatch TS-WRLP, I-O Data Device, Inc., Ishikawa, Japan). Mice behaviors were recorded from the day before treatment to day 4. The nesting material was reset daily at 9:00 h. Nesting materials were disentangled and placed in the center of the cage floor, and the nest consolidation score was assessed 2, 4, 6, 8, 12, 16, and 24 h after placing the materials. On day one, the time when the mice were returned to the cage after surgery or anesthesia was set as the reset time for nest materials. The nest consolidation score was assessed on a 5-point scale: 1 = no nesting sites have been formed and the mouse does not use nesting material; 2 = no nesting sites have been formed, but the mouse uses nesting material; 3 = nest sites have been formed, but some nesting material has not been collected and the nest shape is incomplete and flat; 4 = nest sites have been formed and the nest is cup-shaped; 5 = nest sites have been formed and the nest form is incomplete dome- or dome-shaped (Figure 2). The nest consolidation score was modified with reference to Hess et al. [19].

#### 2.4.4. Burrowing Behavior

Mice burrowing behaviors were also recorded using a network camera. Mice behaviors were recorded from the day before treatment to day four. The water bottle containing glass pellets was reset daily at 5:00 p.m. The latency to burrow was defined as the removal of more than three glass pellets from the device within 10 s [15,20]. The latency to burrow was measured in seconds.

#### 2.4.5. Core Body Temperature

To investigate the effect of treatment on the body temperature of mice, a small temperature measurement device was inserted into the abdominal cavity at the end of the surgery in mice in the surgery + anesthesia + analgesia group. The core body temperature of the mice was recorded with the device from post-surgery to the end of the experiment, and the device was collected at the time of dissection. The data recorded on the device were collected using a FeliCa reader (PaSoRi RC-S380; SONY, Tokyo, Japan) and a dedicated software program (KISSEI COMTEC, Nagano, Japan). The core body temperature of mice was analyzed from 1 to 24 h after surgery.

### 2.5. Histological Assessment of the Stomach and Duodenum

Mice were incised in the abdomen under deep anesthesia (vaporizer concentration dial set at 4%) with isoflurane; the abdominal aorta and posterior vena cava were cut, and cardiac arrest was confirmed, and gross necropsies were performed. Histological assessment was performed on similar sections of the stomach and duodenum of all animals. Tissue sections of the stomach and duodenum were stained with hematoxylin and eosin.

### 2.6. Statistical Analysis

All statistical analyses of data of the remaining 21 mice were performed using Prism 9 (GraphPad Software, San Diego, CA, USA). The differences in the changes in body weight, changes in food intake, and nest consolidating scores between groups were compared using repeated measures two-way ANOVA. Tukey test was conducted as a post hoc test. Kaplan–Meier survival analysis was used to examine the distribution of latency to burrow. The log-rank test was conducted to test whether latency to burrow was statistically different between groups. The body temperature data were compared using the one-way ANOVA. The Tukey test was conducted as a post hoc test. Data are presented as mean ± SE. In all the tests, the significance was set at *p* < 0.05.

## 3. Results

One mouse in the anesthesia + analgesia group was euthanized since it was dying 24 h after the administration of analgesics. The data of one euthanized mouse and two mice (one in the control group and one in the surgery + anesthesia + analgesia group) that were misidentified were excluded from the analysis, except for the necropsy findings and histological evaluation of the stomach and duodenum (the control group: n = 7; the anesthesia + analgesia group: n = 7; the surgery + anesthesia + analgesia group: n = 7). Autopsy and histological evaluation of stomach and duodenum were performed in all individuals (the control group: n = 8, the anesthesia + analgesia group: n = 8, the surgery + anesthesia + analgesia group: n = 8).

### 3.1. Changes in Food Intake

**Figure 3:**
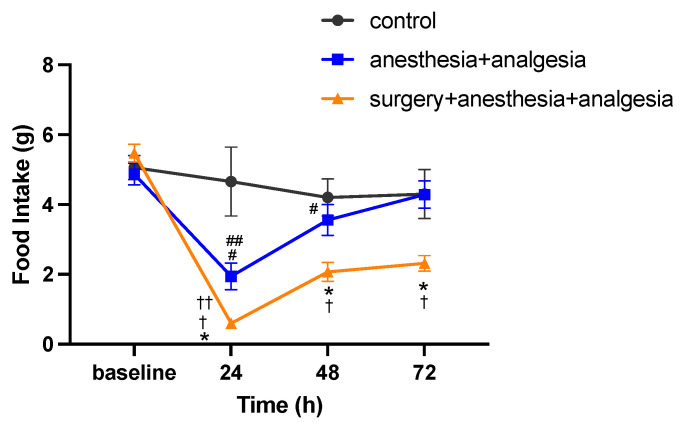
Comparison of postoperative changes in food intake (mean ± SE). *, value differs (*p* < 0.05) between the anesthesia + analgesia and the surgery + anesthesia + analgesia groups. #, value differs (*p* < 0.05) between baseline and 24, 48 h in the anesthesia + analgesia group. ##, value differs (*p* < 0.05) between 24 and 72 h in the anesthesia + analgesia group. †, value differs (*p* < 0.01) between baseline and 24, 48, 72 h in the surgery + anesthesia + analgesia group. ††, value differs (*p* < 0.01) between 24 and 48, 72 h in the surgery + anesthesia + analgesia group. The control group: n = 7, the anesthesia + analgesia group: n = 7, the surgery + anesthesia + analgesia group: n = 7.

The baseline food intake was 5.1 ± 0.3 g in the control group, 4.9 ± 0.3 g in the anesthesia + analgesia group, and 5.5 ± 0.2 g in the surgery + anesthesia + analgesia group. There was no significant difference in the baseline food intake among the groups. In repeated measures two-way ANOVA, the interaction between group and time was significant (F(6,54) = 4.605, *p* < 0.01). In the simple main effect test, for the surgery + anesthesia + analgesic group, the food intake at 24, 48, and 72 h after surgery was significantly lower than that in the anesthesia + analgesic group (*p* < 0.05). For the anesthesia + analgesic group, the food intake at 24 and 48 h was significantly lower than the baseline value (*p* < 0.05), and the food intake at 72 h was significantly higher than that at 24 h (*p* < 0.05). For the surgery + anesthesia + analgesic group, the food intake at 24, 48 and 72 h was significantly lower than the baseline value (*p* < 0.01), and the food intake at 48 and 72 h was significantly higher than that at 24 h (*p* < 0.01) (Figure 3).

### 3.2. Changes in Body Weight

**Figure 4:**
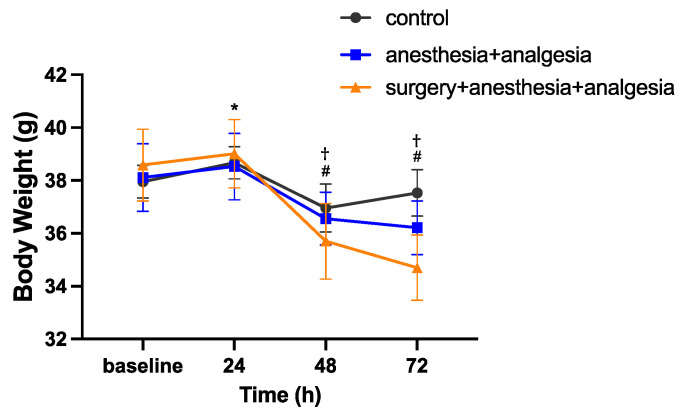
Comparison of postoperative changes in body weight (mean ± SE). *, value differs (*p* < 0.01) between baseline and 24 h in the control group. #, value differs (*p* < 0.05) between baseline, 24 h and 48, 72 h in the anesthesia + analgesia group. †, value differs (*p* < 0.05) between baseline, 24 h and 48, 72 h in the surgery + anesthesia + analgesia group. The control group: n = 7, the anesthesia + analgesia group: n = 7, the surgery + anesthesia + analgesia group: n = 7.

The baseline body weight was 38.0 ± 0.6 g in the control group, 38.1 ± 1.3 g in the anesthesia + analgesia group, and 38.6 ± 1.4 g in the surgery + anesthesia + analgesia group. There was no significant difference in baseline body weight among the groups. In repeated measures two-way ANOVA, the interaction between group and time was statistically significant (F(6,54) = 8.414, *p* < 0.01). In the simple main effect test, for the control group, the body weight at 24 h was significantly higher than the baseline value (*p* < 0.01). For the anesthesia + analgesic group, the body weight at 48 and 72 h was significantly lower than the baseline value (at 48 h; *p* < 0.05, at 72 h; *p* < 0.01), and the body weight at 48 and 72 h was significantly lower than that at 24 h (*p* < 0.01). For the surgery + anesthesia + analgesic group, the body weight at 48 and 72 h was significantly lower than the baseline (*p* < 0.01) and the body weight at 48 and 72 h was significantly lower than that at 24 h (*p* < 0.01) (Figure 4).

### 3.3. Nest Consolidating Behavior

**Figure 5:**
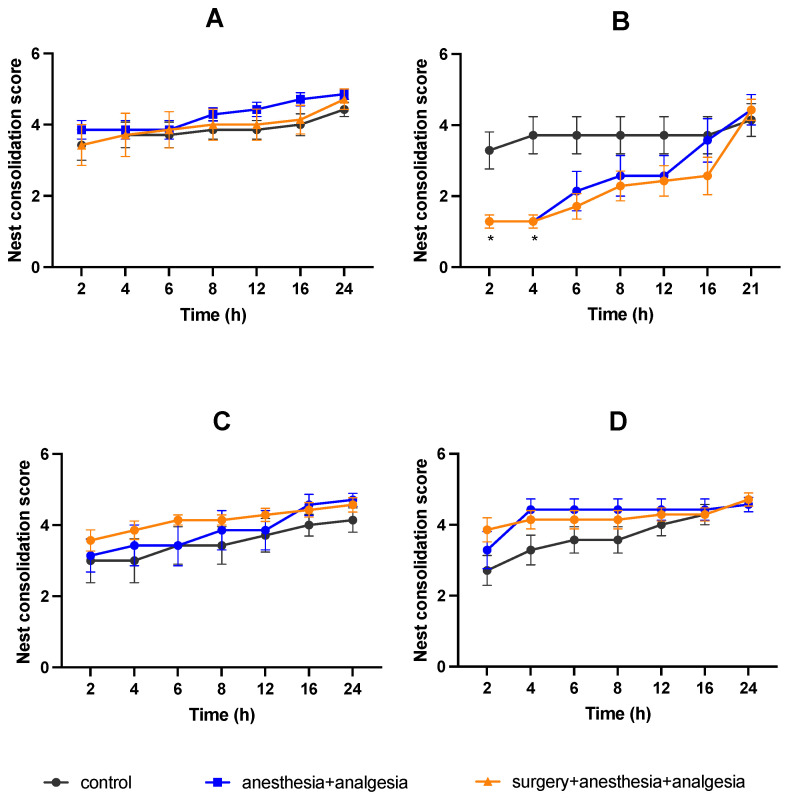
Comparison of the nest consolidation score (mean ± SE): (**A**) Day before treatment. (**B**) Day one. (**C**) Day two. (**D**) Day three. *, value differs (*p* < 0.05) between the control and the anesthesia + analgesia groups. The control group: n = 7, the anesthesia + analgesia group: n = 7, the surgery + anesthesia + analgesia group: n = 7.

There was no significant difference in the pretreatment nest consolidating scores among the three groups (Figure 5A). In the anesthesia + analgesia and surgery + anesthesia + analgesia groups, the nest consolidation score was significantly lower than that in the control group at 2 and 4 h after treatment on day one (Figure 5B). At other points, there was no significant difference in the nest consolidation scores among the three groups (Figure 5B–D).

### 3.4. Burrowing Behavior

**Figure 6:**
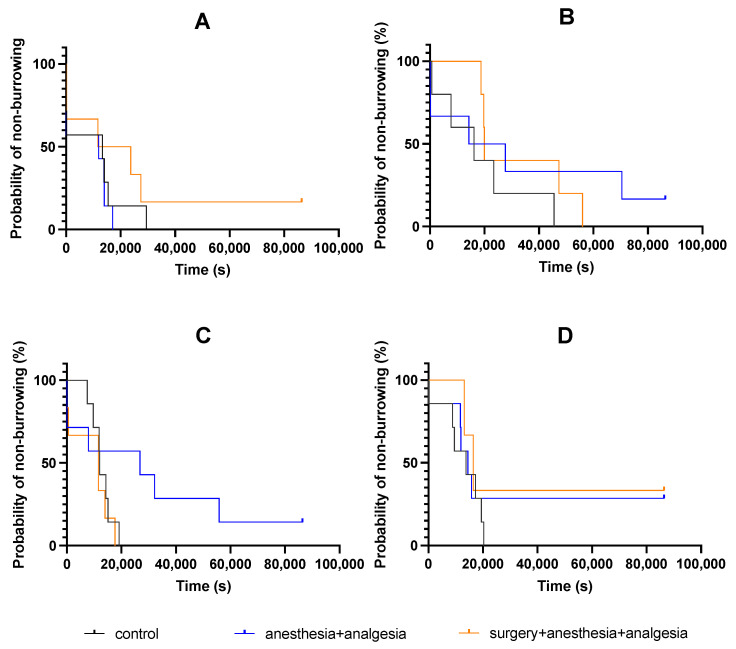
Kaplan–Meier analysis of the latency to burrow: (**A**) Day before treatment. (**B**) Day one. (**C**) Day two. (**D**) Day three. The control group: n = 7, the anesthesia + analgesia group: n = 7, the surgery + anesthesia + analgesia group: n = 7.

There was no significant difference in the latency to burrow on the day before treatment among the three groups (Figure 6A). Furthermore, there was no significant difference among the three groups in the latency to burrow on day one (Figure 6B), day two (Figure 6C), and day three (Figure 6D).

### 3.5. Changes in Core Body Temperature

**Figure 7:**
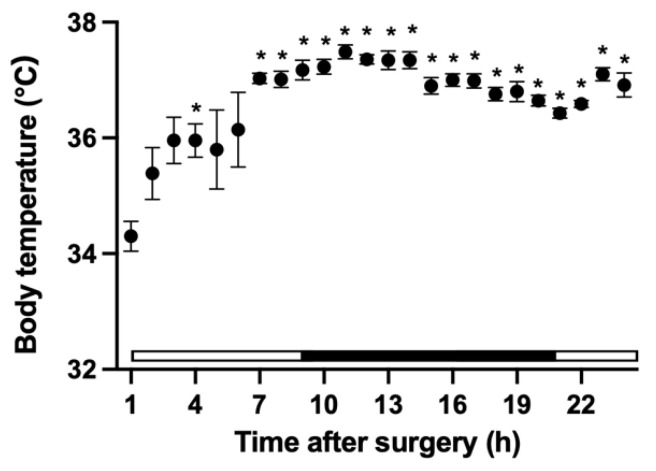
Changes in the core body temperature of mouse after surgery (n = 7). *, value differs (*p* < 0.05) between 1 h after surgery and 4, 7, and 24 h after surgery. Differences in values (*p* < 0.05) between other times are described in the text. The white bar indicates the light period in the animal room and the black bar indicates the dark period.

The core body temperature 1 h after surgery was significantly lower than that at 4 h and 7–24 h after surgery (Figure 7). Furthermore, there was significant difference in core body temperature between 10 h and 21 h, 22 h 11 h, and 20–22 h, 12h and 20–22 h, 13 h and 20 h, 14 h and 20 h, and 16 h and 21 h after surgery.

### 3.6. Autopsy Findings, and Histological Assessment of the Stomach and Duodenum

**Figure 8:**
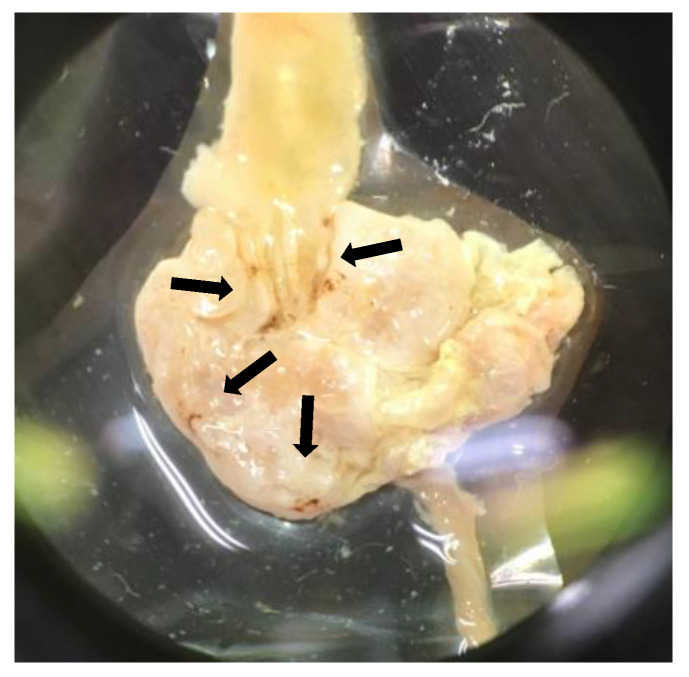
Areas of brown discoloration in the stomach of mouse. The arrows indicate areas of brown discoloration.

At necropsy, areas of brown discoloration were observed in the stomachs of two mice (Figure 8). Both mice were in the anesthesia + analgesia group, and one of the stomachs was from a mouse that had been euthanized due to being moribund during the experiment. However, no morphological abnormalities were found in any of the samples on histopathological evaluation.

## 4. Discussion

As there is no “one-size-fits-all” analgesia for all animal experimental protocols, pain is never relieved completely in many cases. However, the researchers conducting the animal experiments need to be careful to minimize the pain felt by animals. Experimental procedures are among the potential causes of distress in laboratory animals [2]. Although no significant decreases were observed in our study, food intake showed a decreasing trend at 24 and 48 h in the control group, and body weight showed a decreasing trend at 48 h. Due to the high frequency of administration on day one in the protocol of our study, the stress of repeated injections and forced oral administration may have influenced this trend of decreased food intake and body weight [21].

Buprenorphine is an opioid analgesic with spinal and supraspinal site of action [22]. It is one of the most commonly used opioid analgesics in postoperative pain management in mice and rats [8,22]. The administration of buprenorphine may decrease food intake and body weight in postoperative mice [5,8,21,23,24]. Meloxicam is an oxicam-based NSAID. It reduces inflammatory pain by inhibiting prostaglandin synthesis [2]. Inhibition of prostaglandin synthesis reduces the resistance of the gastric mucosa to acidic gastric content, resulting in gastritis, and loss of gastric mucosal cell tissue causes gastrointestinal ulcers. The administration of meloxicam may decrease body weight in postoperative mice [25]. In addition, isoflurane may decrease food intake in mice [26]. The decrease in food intake and body weight in the anesthesia + analgesia group and in the surgery + anesthesia + analgesia group observed in our study, as well as in other studies, might be influenced by the anesthetics and analgesics used. At necropsy, areas of brown discoloration were observed grossly in the stomach of two mice, but no morphological abnormalities were observed in all samples during histopathological evaluation. Studies in which mice were treated with higher concentrations (20 mg/kg) of meloxicam for six days showed no pathological lesions associated with NSAID toxicity, such as gastric ulcers or liver and kidney lesions [27]. It was considered unlikely that the decrease in food intake was due to organic gastrointestinal abnormalities caused by meloxicam, and the relationship between the areas of brown discoloration observed and the decrease in food intake is unknown. 

In the surgery + anesthesia + analgesia group, a small temperature measurement device was implanted into the abdominal cavity of the mouse at the end of the surgical procedure to measure the core body temperature. The core body temperature of mice was significantly low at 1 h after surgery, but it recovered subsequently. The shape of the circadian pattern showed bimodal changes; body temperature increased during the transition from the light period to the dark period, declined once, and increased again from the end of the dark period [28]. It has been reported that intraperitoneal implantation of a radiotelemetry device can compress the internal organs of small rodents, and it may take up to two weeks for weight post-surgery to recover preoperatively [8,28]. In this study as well, weight recovery in the surgery + anesthesia + analgesia group was lower than that in the anesthesia + analgesia group. The implantation of a small temperature measurement device may have acted as a physical stimulation to the gastrointestinal tract of mouse, increasing the reduction in food intake and delaying weight recovery. The anesthetics and analgesics, as well as the intraperitoneal implantation of the temperature measuring device, may have influenced the decrease in food intake in the surgery + anesthesia + analgesia group.

There was no significant difference in latency to burrow among the three groups at any time point. The decrease in the nest consolidation scores at 2 and 4 h after the treatment/surgery on day one was considered to be due to the effect of anesthesia and/or analgesics. Based on the recovery state of the deep body temperature, it was considered that the recovery time from anesthesia most likely affected this variable. It has been reported that under pain, associated with the surgical procedure, the nest consolidating time and the latency to burrow are extended [2,5,14,15,16,17,18]. Our findings support the hypothesis that a multimodal analgesic protocol using buprenorphine and meloxicam may have been effective for analgesia associated with abdominal surgery. However, due to the lack of surgery group without analgesic in our study, we cannot exclude the possibility that this multimodal protocol did not sufficiently eliminate the pain caused by the surgical procedure. Therefore, we cannot exclude the possibility that the decrease in food intake in the surgery + anesthesia + analgesia group included the effects of postoperative pain, and the possibility that postoperative pain did not affect burrowing behaviors and nest consolidating behaviors.

Buprenorphine and indomethacin [8], buprenorphine and carprofen [5,28], morphine and various NSAIDs [29], and fentanyl and paracetamol [7] have been used in multimodal approaches in mice with a combination of opioids and NSAIDs. This is probably the first study to evaluate a multimodal approach using buprenorphine and meloxicam in mice [28,30]. The multimodal analgesic protocol for mice using buprenorphine and meloxicam is expected to have an analgesic effect on pain associated with laparotomy, but caution should be exercised regarding its effects on the decrease in food intake and body weight. Even in the absence of surgical pain, negative effects such as the decrease in food intake and weight loss may be induced by excessive administration of anesthesia and analgesic treatments [5,8]. To ensure the wellbeing of animals, it is necessary to focus on and care not only about the approach to relieve the surgical pain, but also other types of distresses. This does not negate the need to administer analgesics to mice postoperatively, but suggests the need to improve the analgesic agent of choice, dosage and frequency of analgesic administration to minimize negative side effects that may interfere with postoperative recovery in mice. In our study, analgesic treatment was performed up to 72 h after surgery. It may be necessary to improve the duration of analgesic administration, as it has been reported that post-laparotomy pain in mice lasts for about 48 h [31]. Although the dosages of buprenorphine and meloxicam used in this study were generally within the recommended ranges, the results showed that the dosages should be revised to reduce adverse effects such as decreased food intake and body weight [30]. Furthermore, by examining the effects of the physical and social environment on the animals’ postoperative recovery, more appropriate perioperative pain management can be expected in consideration of the wellbeing of animals [20,32,33].

The present study had some limitations. We did not set up a surgical procedure group without analgesics or a surgical procedure group with only buprenorphine or meloxicam. Therefore, it is difficult to evaluate the effect of multimodal analgesia on pain. Only one strain of male mice was used in the experiments. The differences in response due to the differences in mouse strain and sex could not be examined. Bupivacaine was not administered to the anesthesia + analgesia group; the effect of bupivacaine was not reflected in comparison with the surgery + anesthesia + analgesia group.

## 5. Conclusions

Even in absence of surgical pain, the anesthesia + analgesia group presented the same negative effects as the surgery + anesthesia + analgesia group. The multimodal analgesic protocol for mice using buprenorphine and meloxicam is expected to have an analgesic effect on pain associated with laparotomy, but it was not sufficient to prevent food intake and weight decrease. To ensure the wellbeing of animals, it is necessary to focus on and care not only about the approach to relieve pain associated with surgery, but also other types of distresses.

## Figures and Tables

**Table 1:** Drugs administered to each group.

	Treatment	Control	Anesthesia + Analgesia	Anesthesia + Analgesia + Surgery
Day 1	Medetomidine	Water for injection (IP)	0.2 mg/kg (IP)	0.2 mg/kg (IP)
	Atipamezole	Water for injection (IP)	1 mg/kg (IP)	1 mg/kg (IP)
	Isoflurane	–	1.5–2.5%	1.5–2.5%
	Buprenorphine	Water for injection	0.1 mg/kg (SC) 2 times	0.1 mg/kg (SC) 2 times
	Meloxicam	Water (PO)	5 mg/kg (PO)	5 mg/kg (PO)
	Bupivacaine	–	–	0.5% (drop)
Day 2	Meloxicam	Water for injection (IP)	5 mg/kg (PO)	5 mg/kg (PO)
Day 3	Meloxicam	Water for injection (IP)	5 mg/kg (PO)	5 mg/kg (PO)

## Data Availability

Not applicable.
